# Case Report: Rare case of anterior cruciate ligament reconstruction in a 26-year-old female paraplegic patient: technical considerations and rehabilitation outcomes

**DOI:** 10.3389/fsurg.2025.1620241

**Published:** 2025-12-04

**Authors:** Abdullah Raizah

**Affiliations:** Department of Orthopedics, College of Medicine, King Khalid University, Abha, Saudi Arabia

**Keywords:** ACL injury, ACL reconstruction, spinal cord injury, paraplegia, rehabilitation

## Abstract

**Background:**

Anterior cruciate ligament (ACL) injuries in paraplegic patients are rare and pose unique challenges in treatment planning. Functional knee stability is critical for rehabilitation, especially in those regaining partial mobility.

**Case presentation:**

A 26-year-old female with flaccid paraplegia following polytrauma developed symptomatic right knee instability. MRI (1.5 T) confirmed a complete ACL rupture. She underwent ACL reconstruction using an 8.5 mm graft combining ipsilateral hamstring and contralateral semitendinosus tendons. Arthroscopic reconstruction was performed via an anteromedial portal, with femoral suspensory fixation and tibial bioabsorbable screw. A tailored rehabilitation program incorporating neuromuscular stimulation led to progressive improvement in quadriceps strength (MRC Grade 2–4) and gait function.

**Conclusion:**

ACL reconstruction can be a viable option in selected paraplegic patients with partial mobility recovery when mechanical instability impedes functional progress. This case underscores the importance of individualized surgical and rehabilitation strategies in this complex population.

## Introduction

Road traffic accidents (RTAs) are a leading cause of illness and death worldwide, responsible for over 1.3 million deaths and an estimated 50 million injuries each year (1). The burden of RTAs mainly affects low- and middle-income countries, where young and economically active populations are most impacted (2). In Saudi Arabia, motor vehicle crashes remain a major public health issue, accounting for 20% of hospital admissions and 81% of hospital deaths (3). These injuries often cause complex trauma, including spinal cord injuries (SCIs) and related musculoskeletal damage, which further complicate patient recovery (3).

Given the frequency of traumatic injuries in RTAs, it is important to consider the impact of co-existing injuries, such as anterior cruciate ligament (ACL) ruptures (1). Although ACL injuries are common among young and active individuals, their occurrence in paraplegic patients with SCIs is relatively rare and presents unique challenges (1). Functional knee stability remains essential for rehabilitation, especially in paraplegic patients who regain partial mobility, as it directly influences their ability to engage in assisted walking or weight-bearing activities (1, 2).

There is limited evidence regarding the outcomes of ACL reconstruction in paraplegic patients, especially its role in enhancing functional mobility despite significant neurological impairment (2). This case report showcases a successful surgical intervention, tailored rehabilitation, and long-term recovery in a paraplegic patient who regained partial mobility. While ACL reconstruction is well-documented in the general population, literature on its use in individuals with spinal cord injuries remains limited. This report describes a paraplegic female patient with an SCI, focusing on the surgical approach, rehabilitation strategies, and the importance of knee stability in supporting functional recovery. We suggest that ACL rupture in partially recovered paraplegic patients may be an under-recognized obstacle to walking, particularly when muscle strength alone does not explain gait instability. Recent studies continue to point out the scarce reporting of ligament injuries in neurologically impaired groups, with few documented cases of bilateral or complex cruciate ligament involvement.

## Presentation of case

A 26-year-old woman, previously healthy, was involved in a motor vehicle accident on August 5th, 2020, while she was a back-seat passenger and not wearing a seat belt. The accident caused polytrauma, and she was transported to the hospital by ambulance. Upon arrival, she was managed according to Advanced Trauma Life Support guidelines (3) and found to be hemodynamically stable with a Glasgow Coma Scale (GCS) score of 15/15 (4). A thorough evaluation revealed multiple rib fractures (2nd, 3rd, 4th, 5th, 9th, and 10th ribs on the left side with hemopneumothorax and the 9th rib on the right side with mild hemopneumothorax), managed with the insertion of a chest tube on the left side.

Abdominal examination revealed a soft, deep perineal wound measuring 5 cm by 5 cm in the ischiorectal region, with intact rectal tone. The wound was dressed, and the patient was started on broad-spectrum antibiotics along with a tetanus shot. She presented with complete flaccid paraplegia and a deformity in the right leg, which was stabilized using a splint. A pan-CT scan confirmed the injuries, including a T8 vertebral body fracture, sacral fracture, ruptured bladder, and displaced right tibial shaft fracture. The patient underwent emergency surgery on the same day, including a laparotomy with ileostomy and perineal wound debridement by the general surgery team. Bilateral percutaneous ureterostomies were performed by the urology team, with plans for bladder reconstruction at a later stage. The patient developed a wound infection at the laparotomy site, delaying spinal fixation for about six weeks. Once the infection was resolved, she underwent posterior spinal decompression, fixation, fusion, and bone grafting from T4 to T11. Orthopedic management included open reduction and internal fixation of the right tibial shaft fracture using plates and screws. The left clavicle and scapula fractures were treated non-operatively.

**Figure 1:**
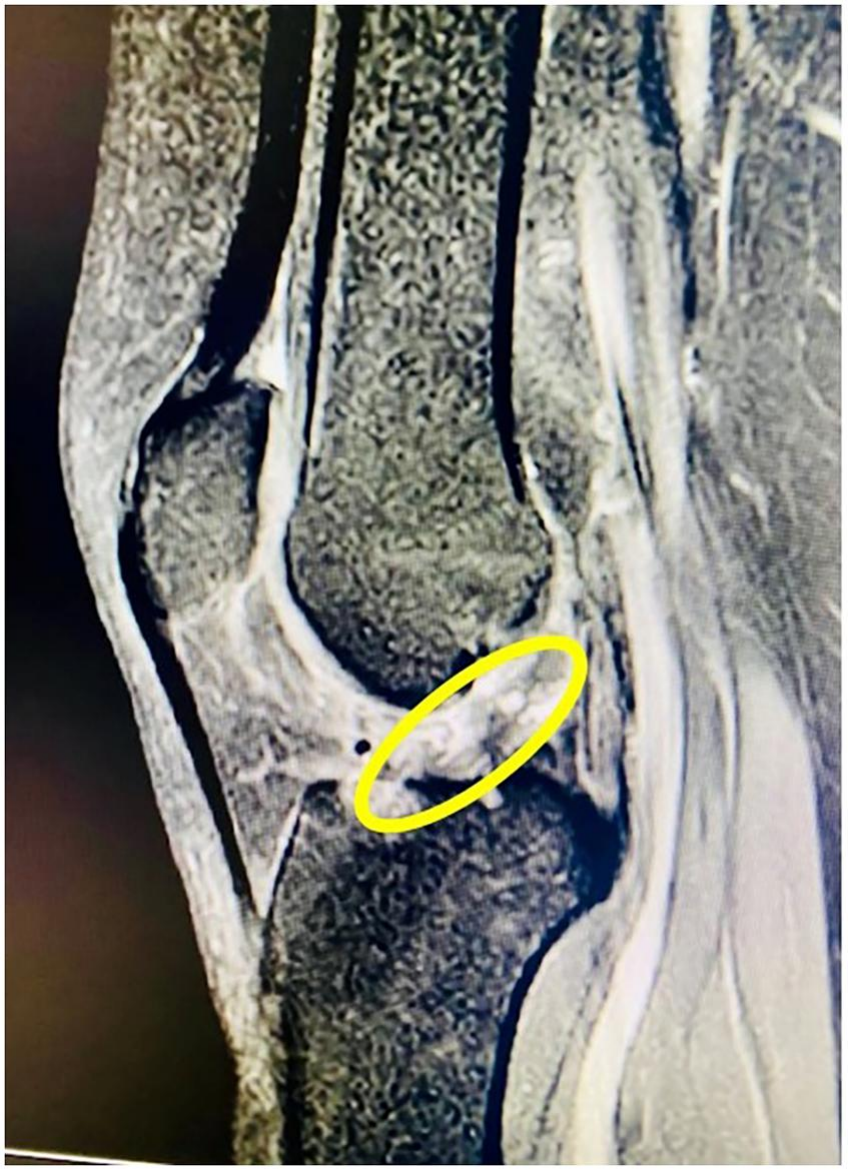
Pre-operative MRI showing complete ACL rupture in a paraplegic patient.

**Figure 2:**
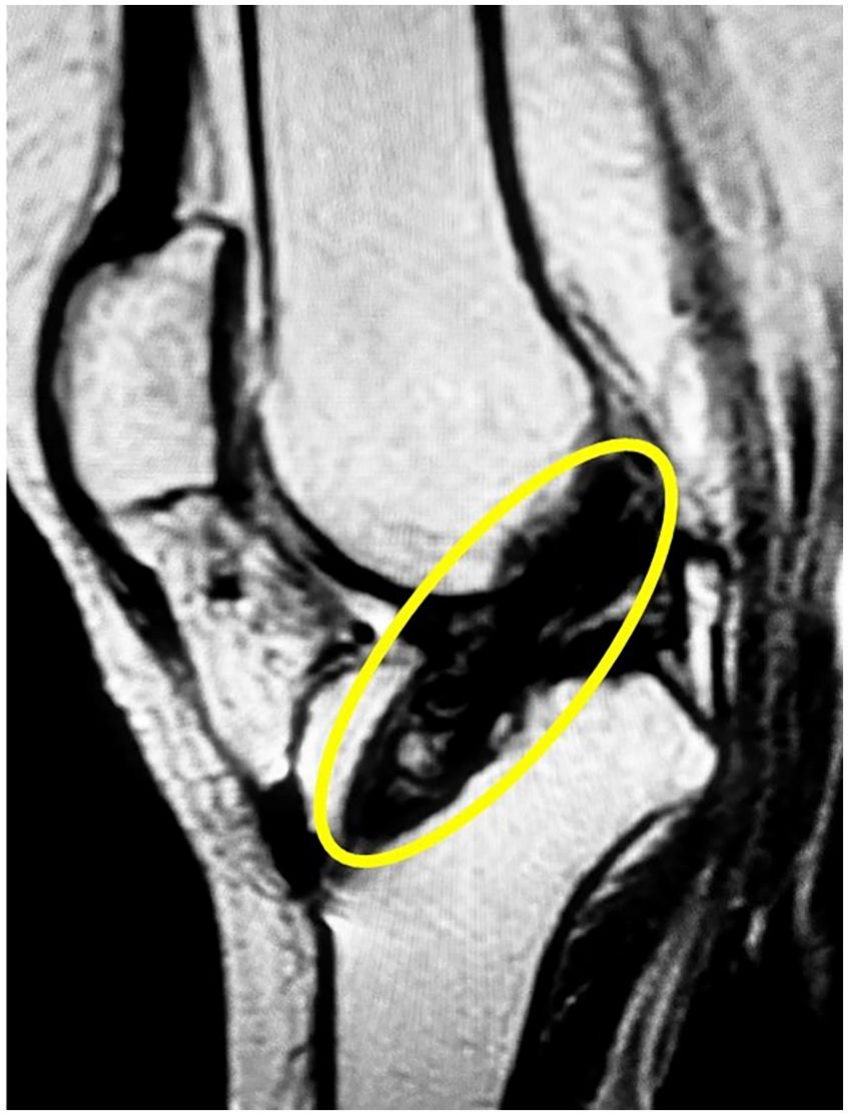
Post-operative MRI demonstrating successful ACL reconstruction with intact graft alignment.

Following her recovery from initial injuries, the patient underwent long-term physiotherapy and rehabilitation for her spinal cord injury. Her paraplegia improved significantly, allowing her to stand and take assisted steps with a specialized walker. However, she reported instability in her right knee, which often gave way and hindered her walking progress. Clinical examination confirmed a complete ACL rupture in the right knee, later verified by magnetic resonance imaging (MRI). Physical exam showed moderate quadriceps atrophy, graded as Goutallier Grade 2. Instrumented laxity testing with the KT-1000 arthrometer indicated 5.2 mm of anterior tibial translation compared to the opposite side, confirming mechanical instability. MRI, performed with a 3 mm slice thickness, revealed high signal intensity on T2-weighted sequences consistent with mucoid degeneration. During diagnostic arthroscopy, about 45% of the ACL and 40% of the PCL fibers showed fraying and were debrided, while remaining ligament fibers were preserved to maintain residual tension and joint proprioception. Pre-operative imaging was crucial for assessing the ACL injury and planning the surgery, while post-operative imaging confirmed accurate graft placement and alignment with surrounding structures. These images validated the surgical outcome and provided a clear assessment of graft integrity. Figures include pre-operative MRI showing the ACL rupture (Figure 1) and post-operative (1-year) imaging demonstrating a successful, intact, and functional ACL (Figure 2).

Given the ACL instability, a thorough discussion was held with the patient and her family regarding treatment options. A specialized brace for ACL deficiency was offered but was poorly tolerated by the patient, who expressed a strong preference for surgery. It was explained that post-surgical physiotherapy and rehabilitation would be challenging due to her weakened lower limb muscles, and all potential complications, including graft failure, were discussed in detail. The patient chose to undergo ACL reconstruction. Preoperative quadriceps strength was assessed as Grade 2 on the Medical Research Council (MRC) scale, indicating movement with gravity eliminated but no active leg lift against gravity. However, this assessment was made in the context of an improving neurological profile and progressive gains during inpatient rehabilitation. Objective knee instability was repeatedly demonstrated through positive Lachman's, anterior drawer, and pivot shift tests, along with MRI-confirmed complete ACL rupture. These findings indicated true ligamentous insufficiency rather than instability caused solely by muscle weakness. The patient had already achieved limited assisted ambulation but continued to experience unpredictable episodes of knee giving way, unresponsive to bracing. Therefore, ACL reconstruction was offered to restore joint stability and enable functional progression, supported by her potential for neuromuscular recovery and high motivation for post-operative rehabilitation.

The surgical technique used an ipsilateral hamstring autograft, supplemented by a contralateral semitendinosus graft, to achieve an 8.5 mm diameter and provide the necessary stability for the patient's weakened lower limb muscles. Suspensory fixation was applied on the femoral side, and a bioabsorbable screw was used for tibial fixation. Choosing a graft of this size was based on evidence linking diameters of 8 mm or more to significantly lower failure rates, especially in individuals with compromised muscle strength or high functional demands. This approach aligns with research emphasizing the importance of graft size in ensuring long-term durability and stability in ACL reconstruction.

## Rehabilitation protocol and progress

Post-surgery, the patient followed a structured and progressive rehabilitation plan tailored to her specific functional limitations caused by paraplegia. The rehabilitation targeted overcoming spasticity-related challenges unique to paraplegic patients. Neuromuscular electrical stimulation (NMES) was incorporated into her physiotherapy to support muscle activation, especially in the quadriceps and hamstrings, and to encourage optimal graft integration (5). This method, combined with gradual weight-bearing activities, addressed her lower limb weakness and aimed to restore dynamic stability (6). Exercises were adjusted to accommodate reduced proprioceptive feedback while focusing on postural control and gait mechanics. During the first four weeks, she performed isometric exercises for the quadriceps and hamstrings to boost muscle activation and prevent atrophy. By the eighth week, she advanced to partial weight-bearing activities with a walker, emphasizing gradual load increase and gait training. At 16 weeks, she showed significant improvements in dynamic balance, allowing her to stand unassisted for short periods. By six months post-surgery, she transitioned to assisted walking, with notable gains in gait stability and coordination. This step-by-step approach, adapted to her recovery progress, underscored the importance of personalized physiotherapy in optimizing functional outcomes and restoring mobility in paraplegic patients undergoing ACL reconstruction.

At follow-up visits, the patient showed significant improvement. Her right knee was stable on clinical tests, including Lachman's, anterior drawer, and pivot shift tests, with KT-1000 arthrometer readings confirming less than 3 mm of anterior tibial translation (6). By two years after surgery, the patient's Knee Injury and Osteoarthritis Outcome Score (KOOS) (7) increased from 45 before surgery to 78, indicating a 73% improvement in functional mobility and a noticeable reduction in knee-related symptoms. Objective measures, such as the increase in quadriceps strength from Grade 2 to Grade 4 on the MRC scale, further supported the positive progress of recovery. These improvements allowed the patient to walk with assistance more stably and confidently, highlighting the long-term benefits of a customized rehabilitation plan. Additional objective evidence included quadriceps muscle strength improving from Grade 2 to Grade 4 on the Medical Research Council (MRC) scale over six months. The patient's KOOS score improved from 45 pre-operatively to 78 at two years, reflecting better knee function and less pain. No complications were reported during or after rehabilitation. By the two-year follow-up, the patient expressed satisfaction with the surgical results, reporting no changes in ACL function and achieving sufficient stability to support her assisted walking.

## Discussion

This case highlights the successful management of a rare presentation of ACL rupture in a paraplegic patient with significant polytrauma from a motor vehicle accident. The patient presented with complete flaccid paraplegia, requiring comprehensive surgical interventions such as spinal fixation and tibial fracture repair. Over time, her paraplegia improved enough that she was able to stand and take assisted steps. However, functional instability of the right knee caused by an ACL rupture hindered her rehabilitation progress. The decision to proceed with ACL reconstruction in paraplegic patients requires careful weighing of surgical benefits against conservative management. In this case, the patient had already achieved partial neurological recovery and limited assisted ambulation but continued to experience recurrent episodes of knee instability that significantly impeded functional gains. Objective clinical tests and MRI confirmed mechanical instability that was not solely due to muscle weakness. Conservative management—including bracing and physiotherapy—was initially tried but proved ineffective. The decision to operate was based on factors such as functional impairment, confirmed structural deficiency, the patient's potential for further recovery, and her strong preference for surgical treatment after informed counseling. Although the surgical technique itself is not new, applying it in this specific neurological context highlights a nuanced clinical decision-making process relevant to similar cases. While bracing remains a non-surgical alternative, its effectiveness is limited in patients with neuromuscular weakness and altered proprioception. In this case, the patient's partial mobility recovery and desire for improved knee stability justified surgical intervention. Contraindications for ACL reconstruction in paraplegic patients typically include complete lower limb paralysis with no expectation of assisted ambulation, poor rehabilitation potential, or other medical conditions that increase surgical risk (7). Ethical considerations also play a role, as ACL reconstruction in patients with uncertain long-term ambulation must be balanced against the invasiveness of surgery and the patient's functional goals. In some cases, ACL reconstruction can enhance rehabilitation outcomes by restoring knee stability, facilitating weight-bearing activities, and preventing secondary joint degeneration (8). This case underscores the importance of addressing co-existing musculoskeletal injuries—even in paraplegic patients—to optimize functional outcomes and quality of life.

### Key learning points

Bilateral cruciate ligament involvement is extremely rare, especially in neurologically impaired patients, and presents distinct diagnostic and treatment challenges. Typical MRI findings, including the “celery stalk” appearance and a narrowed intercondylar notch, can aid in early identification of mucoid degeneration and help plan surgery. In certain cases, arthroscopic partial removal of degenerated fibers combined with notchplasty provides immediate relief of symptoms by decompressing the notch and maintaining ligament function.

### Broader clinical implications

This case indicates that ACL reconstruction may be a feasible option for paraplegic patients who regain partial mobility, providing benefits in knee stability and functional outcomes (9, 10). Functional knee stability is crucial for allowing paraplegic patients to participate in activities such as assisted walking and weight-bearing exercises, which are vital for their overall rehabilitation (11). The patient's insistence on pursuing surgical intervention and her subsequent positive results demonstrate that such procedures can notably improve functional capacity, even in cases of severe lower limb weakness. This case further underscores the importance of personalized treatment plans that consider patient goals, emphasizing the role of shared decision-making in managing complex injuries.

### Surgical technique and rationale

In this case, the decision to use an ipsilateral hamstring autograft augmented with a contralateral semitendinosus graft was motivated by the need to achieve a robust graft size, measuring 8.5 mm in width (10). Preoperative and intraoperative assessments showed that the ipsilateral semitendinosus and gracilis tendons were insufficient to reach the desired graft size. Therefore, harvesting the contralateral semitendinosus was performed to meet the 8.5 mm threshold, which was deemed essential for ensuring long-term graft durability, especially in a paraplegic patient with weakened muscles and impaired proprioception. The decision aimed to prioritize biomechanical stability and functional outcome, despite the increased invasiveness of bilateral harvest. This approach was chosen to maximize knee stability, particularly in a paraplegic patient with weak lower limb muscles (12). Hamstring tendon grafts are preferred for their lower rate of anterior knee pain and reduced donor site morbidity compared to bone-patellar tendon-bone (BPTB) grafts (12). Using a contralateral semitendinosus graft for augmentation resulted in a thicker, stronger graft, which is especially beneficial for patients with compromised muscle strength and stability (13). Suspensory fixation on the femoral side and bioabsorbable screw fixation on the tibial side provided secure graft placement and minimized potential complications (13). This personalized approach highlights the importance of selecting grafts and fixation methods tailored to each patient's characteristics and functional needs (14).

### Comparison to similar cases in literature

The literature on ACL reconstruction in paraplegic patients is limited, reflecting how rare this situation is. However, previous studies on ACL reconstruction in neurologically impaired patients highlight the challenges of rehabilitation and the higher risk of graft failure due to weak muscle strength (14). Compared to other reports, this case shows positive outcomes, with the patient achieving stable knee function and personal satisfaction (15). This supports findings that stress the importance of achieving a large graft size and proper fixation to ensure durability and stability in neurologically impaired patients (16). This case matches findings from similar reports, which show that ACL reconstruction can improve stability in neurologically impaired patients. However, it differs in its graft choice, stressing the need for a strong graft size to make up for weakened muscles. Future studies should look at long-term results in larger groups of paraplegic patients who have ACL reconstruction, focusing on joint wear and preventing secondary injuries. Unlike cases with BPTB grafts, which often lead to more anterior knee pain and osteoarthritis, using hamstring autografts in this case reduced donor site problems and improved patient comfort (17). Additionally, the lack of complications such as infection, graft impingement, or hardware failure shows careful surgical planning and execution.

### Rehabilitation and outcomes

The patient underwent a customized physiotherapy program tailored to her specific needs as a paraplegic with partial mobility. This program focused on strengthening the hamstring and quadriceps muscles, retraining postural control, and enhancing dynamic balance to address her lower limb weakness (18). Weight-bearing exercises and assisted gait training with a specialized walker were gradually introduced to improve functional mobility (19). Post-operative rehabilitation included static and dynamic balance exercises, which are essential for restoring limb alignment control and lowering the risk of future injury.

Objective assessments, such as the KT-1000 arthrometer, confirmed less than 3 mm of anterior tibial translation, indicating excellent graft stability. Clinical tests, including Lachman's, anterior drawer, and pivot shift, further demonstrated stable knee function at each follow-up. The patient's positive outcomes at the two-year mark reinforce the potential benefits of ACL reconstruction in paraplegic patients, with significant improvements in stability and functional capacity.

### Broader implications for practice

This case underscores the importance of considering ACL reconstruction in paraplegic patients who regain partial mobility. Rehabilitation strategies should focus on enhancing functional stability to optimize the patient's ability to participate in assisted walking and other activities. Moreover, this case highlights the need for customized surgical techniques and physiotherapy protocols to address the unique challenges faced by neurologically impaired patients. Further research is necessary to better understand the long-term outcomes of ACL reconstruction in this population, especially regarding the prevention of joint degeneration and graft failure. By analyzing outcomes in a larger group, future studies could develop standardized protocols for the surgical and rehabilitative management of ACL injuries in paraplegic patients.

### Limitations and challenges

Although this case was successful, several challenges warrant consideration. One major limitation is the lack of extended follow-up beyond two years, which restricts conclusions about very long-term graft integrity, joint preservation, and sustained functional improvements. Ongoing clinical monitoring is crucial to confirm the durability of surgical and rehabilitative outcomes. The patient's lower limb weakness created significant rehabilitation obstacles, necessitating an extended and personalized physiotherapy program. Moreover, her paraplegic status required close supervision to ensure proper post-operative care and to prevent complications such as infection or hardware failure. Despite these challenges, the absence of complications and the patient's subjective satisfaction underscore the potential benefits of surgical intervention in carefully selected cases.

## Conclusion

This case shows that ACL reconstruction using an ipsilateral hamstring autograft supplemented with a contralateral semitendinosus graft can offer significant benefits in knee stability and functional mobility for paraplegic patients who regain partial mobility. The success of this case highlights the importance of personalized surgical planning and rehabilitation to optimize outcomes for this unique patient group. It provides a framework for managing similar cases and adds to the limited evidence supporting ACL reconstruction in paraplegic patients.

## Data Availability

The original contributions presented in the study are included in the article/Supplementary Material, further inquiries can be directed to the corresponding author.
